# GERIATRIC WORKFORCE ENHANCEMENT PROGRAMS AS AAA AGE-FRIENDLY INITIATIVES PARTNER

**DOI:** 10.1093/geroni/igad104.2067

**Published:** 2023-12-21

**Authors:** Jennifer Severance, Linda Edelman, Barbara Gordon, Susanna Luk-Jones, Jacqueline Telonidis, Jennifer Morgan

**Affiliations:** University of North Texas Health Science Center, Fort Worth, Texas, United States; University of Utah, Salt Lake City, Utah, United States; University of Louisville, Louisville, Kentucky, United States; University of North Texas Health Science Center at Fort Worth, Fort Worth, Texas, United States; University of Utah, Salt Lake City, Utah, United States; Utah State University - Institute for Disability Research, Policy and Practice, Logan, Utah, United States

## Abstract

The Health Resources and Services Administration funded 48 Geriatric Workforce Enhancement Programs (GWEPs) to partner with community-based organizations in addressing gaps in health care for older adults, promoting Age-Friendly ecosystems, and addressing social determinants of health. This paper discusses three GWEPs collaborative projects with Area Agencies on Aging (AAAs) that deliver community-based programs . The University of Utah’s GWEP, a rural AAA, the Alzheimer’s Association, and the Utah Department of Veterans and Military Affairs provided a hybrid Dementia Caregiver Conference to six counties, reaching 65 in-person attendees and 10 senior centers online. Attendees agreed the content met their educational needs, would positively influence care, and was motivation to form a caregiver coalition. The University of Louisville Trager Institute (Trager), AAA, and Kentucky Coalition for Healthy Communities provided focused regional training and sharing of community resources utilizing the Project ECHO training model for 11 AAA regions, reaching 412 home and community-based services professionals. The University of North Texas Health Science Center, AAA, and emergency management services expanded evidence-based programs in falls prevention, medication safety, and dementia caregiving, serving an additional 23 urban postal codes, rural counties, and over 1,900 individuals. These examples utilize unique community relationships and partnership strategies to identify local needs, maximize older adult and family caregiver engagement, measure outcomes, and achieve GWEP goals to educate diverse, underserved and rural communities.

